# A longitudinal analysis of mental and general health status of informal carers in Australia

**DOI:** 10.1186/s12889-019-7816-8

**Published:** 2019-11-01

**Authors:** Itismita Mohanty, Theo Niyonsenga

**Affiliations:** 1 Health Research Institute, Faculty of Health, University of Canberra, Canberra, Australian Capital Territory 2617 Australia; 2 School of Health Sciences, University of South Australia, Adelaide, 5001 Australia

**Keywords:** Informal carers health, Mental health, General health, Health behaviour and informal carers age

## Abstract

**Background:**

The study investigated the self-assessed mental and general health status of informal carers in Australia. It evaluated the influence of carer’s health behaviours, namely physical activity, smoking and drinking status, along with their social connectedness and workforce engagement on their health status.

**Methods:**

The study used a retrospective longitudinal design using data from the Household Income and Labour Dynamics of Australia survey, waves 5–15 (2005–2015). It included individuals aged 15 years and older from Australian households surveyed over a period of 11 years. The sample consisted of 23,251 individuals. The outcome measures included: mental health, general health and physical functioning domains of the *Short Form 36 Questionnaire*, a widely used multi-dimensional measure of health-related quality of life. Using fixed effects regression and following individuals over time, the analysis took care of the issue of individuals self-selecting themselves as carers due to some predisposing factors such as age, poor health, socioeconomic status and sedentary behaviour.

**Results:**

There were statistically significant carer-noncarer status differences in mental (Beta = − 0.587, *p* = 0.003) and general health (Beta = − 0.670, *p* = 0.001) outcomes. Aging had a modifying impact on carers’ mental and general health outcomes. Older carers coped better with their caregiving responsibilities than younger ones. Moreover, while physical activities had a positive influence on both mental and general health for non-carers, with more activities generating better health outcomes, it only had a modifying impact on carers’ mental health. Furthermore, the study found that moderate levels of social drinking had beneficial modifying impact on carers’ mental and general health.

**Conclusion:**

This study added value to the literature on informal carers’ mental and general health in Australia by identifying some of the protective and risk factors. The study found the modifying effects of carers’ age, health behaviours such as physical activity, smoking and drinking status on their health. Finally, the study identified an apparent beneficial link between moderate levels of social drinking and carer health that needs to be further explored with more targeted future research.

## Introduction

Informal carers are those who have a main role providing care for someone, closely related to them, a friend or a neighbour, primarily in the home environment or outside with a range of physical, mental and end-of-life health conditions, and disability [1, 2]. They make a significant contribution to the care and wellbeing of people with a disability, mental illness, chronic condition, terminal illness and the elderly. In 2015, over 1 in 8 Australians (2.86 million) were estimated to be providing informal care, which was estimated to have a replacement value of $60.3 billion, equivalent to 3.8% of GDP in Australia [2]. Worldwide, the population is ageing, and demographics are changing as a result of decades of declining fertility rates and increasing longevity [3–6]. Social changes have resulted in the breaking of gender stereotypes and changing the role of family with more women in the workforce [3], smaller and dispersed families, lower marriage rates and higher divorce rates [7, 8]. These changes also mean that we are facing an increasing burden of disease and disability with less people available to provide informal support and care. There are rising demands for carers in nursing homes and aged-care facilities, community disability services and for informal carers at home or outside [9]. Under such circumstances, like elsewhere in the world, formal care in Australia has not been able to cope with the required pace for the overall care need and informal carers have assumed a pivotal role in society.

Literature on informal carers’ health has gathered momentum due to the impact of their caregiving on their overall health. The act of providing care by a carer is referred to as caregiving. Providing care for an elderly relative, spouse or a disabled child often restricts the life, social activities, and employment opportunities of the carer. Carers may have less time for leisure [10] or health promoting physical activity [10–12]. Recent evidence increasingly suggests that caregiving is a potential health risk and a chronic stressor that places carers at risk for physical and mental health problems [13–16]. Informal carers are suffering from worse health outcomes than the general population of their age and gender. Indeed, studies have found evidence of impaired health behaviours among carers helping with basic activities of daily living (ADLs) [17–20]. Carers tend to neglect their own health [18] and this impacts on their physical and mental health. Stronger impacts are observed on mental health than on physical health [18].

### Carer socioeconomic demographic characteristics and health

The effects on carer health are further exacerbated by the carer’s age, socioeconomic status, and the availability of informal support [21]. Older carers, people of low socioeconomic status, and those with limited support networks report poorer psychological and physical health than carers who are younger and have more economic and interpersonal resources [21]. Studies have also shown that women carers have worse health outcomes than men [11, 22]. Studies which focused on specific groups, such as women carers in their 40s and older age groups, and/or specific long-term health condition of the recipients, such as Alzheimer’s disease or dementia, have found that the impact of caregiving on carer’s health varies according to the relationship between the carer and the recipient. This gets further compounded by the gender of the carer. A recent longitudinal study among carers of Alzheimer’s disease in Germany observed that providing care for spouse/partner is more damaging for mental health of men and cognitive well-being of women compared to providing care for parents or parents-in-law [23]. Penning et al. (2015) in Canada found that, for women, caring for a spouse or children was more stressful and detrimental to mental health than caring for parents or others [24]. However, there is also evidence that gender differences in caregiving indicators were small to very small in magnitude [13]. A meta-analysis reveals that while women report higher levels of burden and depression, and lower levels of subjective well-being and physical health, they also provide more caregiving hours, help with more caregiving tasks, and assist with more personal care. Therefore, controlling for the gender differences in stressors and resources in statistical analyses reduces the size of gender differences in depression and physical health to levels that are observed in non-caregiving groups [13].

The magnitude of carers’ suffering from adverse health outcomes depends on the number of hours they spent on caring duties and their level of workforce participation. Kenny et al. (2014) have identified that the combination of high levels of caregiving with full-time workforce participation increases the risk of negative physical and mental health outcomes particularly in female carers [22]. On the contrary, in Japan, the impact of high intensity caregiving on carers’ mental health gets aggravated by non-workforce participation and the initial mental health status of the carer, while there is no impact observed on the serious mental distress of irregular employees [25]. Therefore, high intensity caregiving does not suit well either with full-time workforce participation or with non-workforce participation in influencing physical and mental health of carers. Farrugia et al. (2018) found as well that, in Australia, women carers reported worse mental health than non-carers while they were also, more likely to be working part-time or not be working at all [26]. Thus, there is a clear need for understanding the potentially modifying or protective effects of workforce participation on carer health.

Studies suggest that social support realised through family and friends enhances health and wellbeing of people irrespective of their stress levels or protects people from the pathogenic effects of stressful events [27–30]. Consequently, it will be intriguing to assess if the carers’ social support network exhibits any modifying or protective influence on their health.

### Carer health behaviour and health

Although there is growing international literature on both the physical and mental health of carers, there is little focus on the possible pathways through which caregiving influences carer’s health. Many reported studies are restricted in their scope and ability to understand the connection between caregiving and carer health in more detail. Vitaliano et al. (2003) identifies two pathways by which carer’s chronic stress can impact their health [21]. One pathway could be that chronic stress leads to psychosocial distress and increases stress hormones that may contribute to disease development. The other pathway that leads to chronic stress may promote carer’s unhealthy behaviours such as, drug and alcohol abuse, smoking, poor nutrition, and sedentary lifestyle, that are often associated with physical and mental health problems [20]. In an Australian study of women aged 50 years or more, carers reported higher symptoms of depression, anxiety and stress than non-carers [26]. This study also, reported significantly lower participation in health promoting activities and physical activities [26]. Consequently, there is a need to understand the link between caregiving, carer’s healthy behaviour and health. Also, previous studies in Australia have not discussed in detail what might be the other contextual motivating factors that modify or protect carer’s health.

### Rationale and study objectives

Few studies have examined carers’ health using a longitudinal analysis that follows an individual through their pre-to-post caregiving time. Carer’s health status prior to the commencement of caregiving is an important confounder since pre-existing health disparities (beyond those accounted for by age and sex) between carers and non-carers might give rise to misleading conclusions in a cross-sectional analysis. A range of pre-existing factors such as poor socioeconomic status, disengagement with workforce, age, poor health, poor dietary or sedentary behaviour might predispose a person to become a carer [21, 22]. An Australian study found that middle-aged women in poor health tend to be selected into caregiving roles, probably because they are less and/or not at all engaged with the paid workforce. Poor health and disengagement from the paid workforce continue even when caregiving stops [31]. Overall, the literature lacks evidence on understanding the association between a person’s caregiving status (active carer/non-carer), health behaviour, other contextual factors and health in Australia using population-based longitudinal data. Filling this research gap would help in identifying risk and protective factors for carers’ health, also in designing and implementing interventions that enhance protective factors and in reducing the impact of these risk factors on carers’ health.

This study investigated the self-assessed mental and general health status of informal carers, compared to non-carers, in Australia using retrospective analysis of longitudinal survey data. Using this approach, we studied the health of individuals over time through their pre-to-post caregiving years. Such longitudinal analysis inherently eliminated the effect of gender, personality traits and other individual level behavioural predispositions that did not change over time. In other words, a longitudinal fixed effects regression analysis controlled for the pre-existing health disparities in individuals and other factors such as their pre-existing poor socioeconomic status, disengagement with workforce, age, poor dietary or sedentary behaviour, that might have predisposed a person to become a carer. Additionally, we controlled for the effect of variation in age and socio-economic status on the health status of carers over time. The study focused on understanding the potentially modifying or protective effects of carer’s workforce participation and the level of social engagement on their health.

The primary objectives of this study were to assess if caregiving status had an influence on overall health status and to understand whether carer’s health behaviour, level of social engagement and employment status had modified impact on their health, controlling for the effect of other confounders in the model such as age and socio-economic status. Using a retrospective longitudinal study design and following the trajectory of an individual carer’s life through their pre-to- post caregiving years, this study examined the influence of caregiving status and individual level health behaviours, such as alcohol use, smoking and physical activity level, employment status and level of social engagement, on their self-assessed mental and general health. It is anticipated that this may provide the foundation for designing intervention programs for carers to improve their health outcomes.

## Methods

### Data source

The data for this study came from the Household Income and Labour Dynamics of Australia (HILDA) survey. HILDA is a major largescale population based longitudinal survey of Australian households available over a period of 16 years [32]. The survey started in 2001 and follows the lives of more than 17,000 Australians each year. It collects information on economic and personal well-being, labour market dynamics, family life, household and family relationships, income and employment, health and education. Our analysis was restricted to the waves 5–15 (2005–15) spanning a period of 11 years. Our study sample contained Australians aged 15 years and over who completed the self-completion questionnaire (SCQ) that was introduced in HILDA in 2005 [32]. We used an unbalanced panel that included all respondents and enumerated persons who responded to the SCQ and were available at least in two waves (between wave 5 and 15) as opposed to a balanced panel that would only include information on individuals consistently responding in all waves. For more information on HILDA survey sampling methodology including their eligibility criteria, retention rate, missing data, weighting and clustering information please refer to the HILDA User Manual and HILDA project discussion paper series #1/15 [32, 33]. Our study sample consisted of 121,410 responses (or person-years of observation) of 23,251 individuals after excluding the missing values on key dependent and independent variables. On average individual responses were available across 5 waves with a maximum of 11 waves. Table 1 presents information on sample sizes, number of carers and their age across the waves.

**Table 1:** Sample size, Number of Carers and Carers’ Age by Wave [5–15]

Wave	Sample size	Number of Carers	Carer’s Mean Age	Carer’s Minimum Age	Carer’s Maximum Age
Wave 5 (2005)	9843	670	49.9	15	89
Wave 6 (2006)	9328	655	50.5	15	91
Wave 7 (2007)	9076	566	51.4	15	90
Wave 8 (2008)	8971	610	51.3	15	88
Wave 9 (2009)	9200	593	51.6	15	89
Wave 10 (2010)	9950	642	51.4	15	89
Wave 11 (2011)	12,980	908	51.6	15	90
Wave 12 (2012)	12,903	989	51.5	15	90
Wave 13 (2013)	12,986	908	52.9	15	92
Wave 14 (2014)	13,143	975	52.9	15	90
Wave 15 (2015)	13,030	900	52.2	15	89

Note: Table 1 presents sample size, number of carers and average age of carers in each wave of HILDA data

### Measures

The outcome variables included self-reported health, the scores on the *Short Form 36 Questionnaire* (SF-36), a widely used multi-dimensional measure of health-related quality of life, using data available in HILDA surveys. The SF-36 is a multi-purpose, short-form health survey that measures health across eight domains of physical and mental health, that is comprised of 36 questions that focus on general health, physical functioning, role physical, bodily pain, vitality, social functioning, mental health and role emotional [34]. We used general health, physical functioning and mental health domains as our outcome variables. Scores on each of the SF-36 domains are standardised and component scores range from 0 to 100. Higher scores indicated better health [34]. The SF-36 is a generic measure, as opposed to one that targets a specific age, disease, or treatment group.

The SF-36 has proven useful in surveys of general and specific populations, comparing the relative burden of diseases, and have been translated in more than 50 countries as part of the International Quality of Life Assessment (IQOLA) Project; and studies of reliability and validity [35]. Studies of the SF-36 general health domain have yielded content, concurrent, criterion, construct, predictive evidence of validity and test-retest reliability with levels of internal consistence between 0.59–0.79, and estimates of reliability about 0.84 for the general health domain [35, 36].

We have included in our regression model a range of explanatory variables that were expected to have an influence on the general, physical or mental health of an individual over time along with his/her caregiving status. The caregiving status of the individual, the main factor of interest in this study, was defined in each wave as a dichotomous variable, where 1 represented the individuals who actively cared for a household member or non-resident individual due to a long-term health condition or elderly status (carers), and 0 represented those who were not active carers (non-carers). Carers specifically identified themselves as active carers in HILDA survey. We also included the number of hours a person spends on caregiving duties per week as a continuous variable irrespective of their caregiving status in each wave. Therefore, non-carers (or caregiving status = 0) also reported the time they spent on some type of caregiving in the household over a week even if they had not identified themselves as active carers of someone needing long-term care in each wave. On average, non-carers spent much less time on caregiving duties than carers (0.38 of an hour compared to 14.5 h a week). Table 2 presents the other time-dependent predictors (i.e. any predictor whose value for a given individual may have changed over time) such as sociodemographic variables, social network, smoking and drinking status, physical activity status, any major adverse health event occurred in the previous year and the weekly time (hours) spent on volunteering and charity work. Along with the daily/weekly smoking status of an individual, we included the number of cigarettes a person usually smokes per week divided by 10 as an overall control variable. All these variables included in the model are fully detailed in the 2017 HILDA User Manual – Release 16 [32].

**Table 2:** Descriptive Statistics of the Variables over time (Waves 5–15)

Variable	Mean/Percent (total responses =121,410, N = 23,251)	Minimum Average across waves	Maximum Average across waves
SF36 Mental Health Component Score	74.41 (17.08)	73.61	74.99
SF36 General Health Component Score	68.46 (20.94)	67.25	69.69
SF36 Physical Functioning Component Score	83.90 (22.92)	83.43	84.60
Household financial year gross total income ($)	108,261.2 (102,164.1)	81,929.9	126,464.0
Age	44.17 (18.54)	43.4	44.9
Life events in past year: Serious personal injury/illness (# of events)	8.79	7.59	9.75
Time spent (hrs/mins) per week Volunteer/Charity work	0.99 (3.48)	0.9	1.1
Employment Status			
Employed - works 35 hours a week (Base Category)	42.46	40.82	44.34
Employed - works less than 35 hours a week	20.78	20.33	21.36
Unemployed, retired, home duties, students & others	36.76	34.99	38.20
Carer Characteristics		40.82	44.34
Actively cares for a household member/non-resident due to long-term health condition, elderly (%)	6.93	6.24	7.66
Time spent in (hrs/mins) per week Caring for disabled/elderly relative (*n* = 8416)	14.55 (26.85)	11.47	17.52
Carers’ Age (n = 8416)	51.65 (15.89)	49.91	52.86
Alcohol drinking status			
Never drink/No longer drink (Base category)	18.12	16.17	20.16
Drink only rarely	22.82	21.92	23.53
Drink 2/3 days per month	12.61	11.40	13.35
Drink 1/2 days per week	18.91	18.12	19.52
Drink 3 or more days per week	27.54	25.13	30.65
Standard drinks per day			
Don’t drink (Base Category)	18.12	16.17	20.16
1 to 2 standard drinks	42.82	41.92	44.67
3 to 4 standard drinks	21.24	20.50	22.08
5 and more standard drinks	17.82	16.96	19.20
How often participate in Physical Activities?			
Not at all (Base Category)	10.52	9.17	11.18
Less than once a week	15.81	14.65	17.18
1 to 2 times a week	23.66	22.72	24.41
3 times a week	16.07	15.59	16.79
More than 3 times a week	33.94	33.09	35.39
How often get together socially with friends/relatives?			
Every Day	3.91	3.08	4.73
Several times a week	23.65	21.92	26.37
About once a week	31.61	30.99	32.60
1 to 3 times a month	29.89	28.02	32.07
1 or 2 times every 3 months	5.78	4.64	6.46
Less often than once every 3 months (Base Category)	5.17	4.30	5.70
Smoking Status			
Never smoked/No longer smoke (Base Category)	81.39	77.65	83.59
Smoke less often than weekly	1.53	1.36	1.82
Smoke at least weekly (but not daily)	1.80	1.53	2.26
Smoke daily	15.28	13.34	18.28
Number of cigarettes usually smoked each week	14.95 (42.47)	12.9	17.2

Note: The Table 2 presents summary statics of the variables used in the fully-adjusted fixed effects models for the SF36 mental and general health outcomes across 11 waves (wave 5–15) of HILDA data

### Statistical methods

We have fitted panel data fixed effects (FE) regression models [37, 38] to study the extent to which the informal caregiving status and caregiving time along with other time-varying independent variables were associated with the self-assessed general, physical and mental health status of individuals over a period of 11 years. A FE model was preferred over a random effects (RE) [39, 40] in this study primarily guided by our research objective. We examined the health impacts of caregiving for an individual by assessing change over time (controlling for pre-existing health status and socioeconomic status) from before caregiving to, in some cases, after caregiving responsibilities were over, that is, including transitions into and out of care-giving status. The FE modelling approach and our research objective were very well supported in this study using the HILDA survey data that exhibits enough longitudinal variation at individual level. Additionally, Hausman tests undertaken independently on the general and mental health component scores justified our preference of FE models over RE, the null hypothesis being that the preferred model is random effects (RE) while the alternative is the fixed effects (FE) [41, 42]. Under the null hypothesis, the difference in estimated coefficients (RE vs. FE approaches) is not systematic. The test results in a chi-square statistic based on the difference in both coefficients and is expected to be small under the null hypothesis.

Increasing individuals’ age was used as the changing time indicator in this analysis and the FE estimator was the weighted mean of the individuals’ slopes. The model controlled for life events in the past year, time spent volunteering, time spent caregiving for disabled/elderly relatives, employment status, alcohol drinking combined with standard drinks per day (alcohol drinking status), physical activity status, social networking status, smoking status and number of cigarettes per week, as well as household gross total income, all included as fixed effects in the analysis. To test the moderation effects of the above-mentioned independent variables on outcomes, the models also included interaction-effects involving the interaction of carer’s status with age, employment status, drinking status, physical activity, social networking and smoking status and tested whether such interaction effects were significant in predicting carers’ health [43, 44].

We fitted separate models for each of the three SF-36 domain component scores. After estimating a series of regression equations on each health measure, we had selected and presented the model that best fits our sample data and answers the research question. Standard errors for clustered errors at the individual level were computed to account for heteroscedasticity and serial correlation of the error terms [45]. Analyses were performed using STATA 14 (XTREG FE) with robust standard errors estimation [46, 47].

## Results

The descriptive statistics of the total responses (i.e. 121,410 observation-years) along with the minimum and maximum average values across 11 waves are presented in Table 2. The average age across all respondents’ observation-years was 44 years. Carers’ age ranged from 15 to 92 years with an average age of 52 years across all waves. The number of carers varied from 566 (wave 7) to 989 (wave 12) (see Table 1), and they constituted nearly 7% of the total observation-years. Those who served as carers, on an average spent 2.7 years on active caregiving responsibilities over the 11 year window. While in active caregiving status, people spent on average 14.5 (Standard Deviation (SD) =26.8) hours a week in caregiving activities, compared to non-carers who spent less than an hour (0.38 of an hour with a SD = 3.6). In total, 42.5% of the respondents were in full-time employment. The alcohol and smoking status revealed 18% of the respondents across all waves were non-drinkers and 81% were non-smokers.

### Carer and non-carer health

**Fig. 1:**
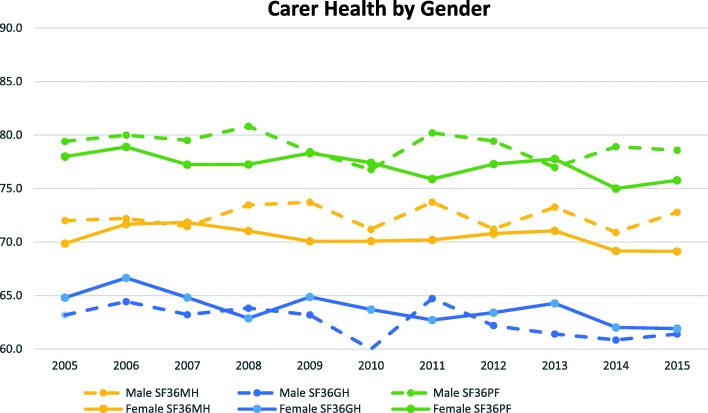
Carer Health Status across the Waves by Gender

**Fig. 2:**
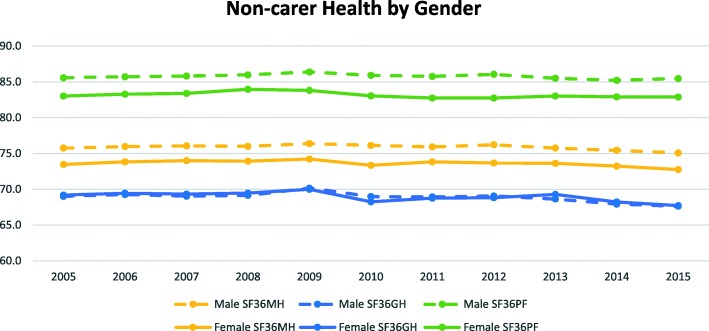
Non-carer Health Status across the Waves by Gender

Figures 1 and 2 present mean values of carer and non-carer’s health status across the waves by gender. Overall, non-carers showed better outcomes than carers in all three health measures. Also, non-carers showed consistently even and slightly declining trends over time with males doing better than females in mental health and physical functioning. Whereas carers showed fluctuating and slightly decreasing trends over the years in all three health measures with the exception of mental health for males, where we see a slightly increasing trend over the years.

Results of the fully adjusted FE models are presented in Table 3 for both SF36 mental (first three columns) and general health (subsequent three columns). In Table 3, the first row presents the impact of *Carer Status* on mental health and general health outcomes. The following section titled *Effects on Non-Carer Status: main terms*, presents the estimates of effects of non-carer’s characteristics such as their age and caregiving time and their health behaviours (physical activity level, smoking and drinking level), employment status and level of social engagement. The section titled *Differences in Carer* versus *Non-carer Status: interaction terms,* presents the non-carer and carer status differential estimated effects. Overall the caregiving status was interacted with other individual characteristics (such as their age and caregiving time, health behaviours, employment status and level of social engagement) to produce *Carer differential effects*. There were statistically significant carer/non-carer status differences in mental health (estimate (Beta) = − 0.587, 95% confidence interval (CI): (− 0.972, − 0.203), *p* = 0.003) and general health (Beta = − 0.670, 95%CI: (− 1.058, − 0.283), *p* = 0.001). On the other hand, there was no significant carer-noncarer status difference in SF-36 physical functioning scores (as shown in Table 4). Consequently, we did not proceed with further analyses for this component. Individuals showed significant disadvantages in both mental and general health outcomes due to being active carers across waves. These carer status disadvantages remained highly statistically significant even after controlling for a range of other household and personal level social, economic, demographic characteristics, smoking, drinking and health behaviour in the model (Table 3). On average, carers reported worse mental and general health scores that are 3 points less than those of non-carers. Additionally, we found that the time spent per week in caregiving duties was negatively related to the mental health score but not to the general health score. The negative influence of time spent on caregiving duties was persistent across carer status levels even though on average people in non-carer’s status spent much less time on caregiving than those in carer’s status (0.38 of an hour compared to 14.5 h a week). Other confounders in the model, namely, household gross income, any serious personal injury/illness in the past year and time spent per week volunteering/charity work had impact on both mental and general health measures in the expected direction in the overall model. The first two of these expected effects were statistically significant, whereas time spent in volunteering/charity work was not significant.

**Table 3:** Fully adjusted fixed effects models separately for the SF36 Mental and General Health components: Estimates of carer status effect, effects of individual characteristics on non-carer status and carer status differential effects (interaction effects)

Variables	SF36 Mental Health Component			SF36 General Health Component		
	Coef.	[95% Conf. Interval]		Coef.	[95% Conf. Interval]	
Carer Status (Yes/No): Actively cares for a household member/non-resident due to long-term health condition, elderly	−3.010***	−5.371	−0.648	−3.106***	−5.378	− 0.834
Effects on Non-Carer Status: main terms						
Non-Carer: Age	−0.009	− 0.039	0.021	− 0.421***	− 0.453	− 0.390
Non-Carer: Time spent in (hrs/mins) per week Caring for disabled/elderly relative	− 0.039***	− 0.065	− 0.012	− 0.003	−0.025	0.018
Non-Carer: Employment Status						
Employed - works 35 hours a week (Reference group)						
Employed - works less than 35 hours a week	−0.560***	− 0.838	− 0.282	−0.706***	− 0.985	−0.427
Unemployed, retired, home duties, students & others	−1.412***	− 1.746	− 1.077	− 1.158***	− 1.498	− 0.817
Non-Carer: Alcohol Drinking Status						
Never drink/No longer drink (Reference group)						
Drink only rarely & 1 to 2 standard drinks	−0.414**	− 0.813	− 0.014	0.259	− 0.129	0.647
Drink only rarely & 3 to 4 standard drinks	−0.818***	−1.385	− 0.250	− 0.195	− 0.744	0.353
Drink only rarely & 5 and more standard drinks	−1.289***	−2.020	−0.558	− 0.208	− 0.915	0.499
Drink 2/3 days per month & 1 to 2 standard drinks	− 0.416	− 0.906	0.074	0.502**	0.015	0.990
Drink 2/3 days per month & 3 to 4 standard drinks	− 0.806***	− 1.352	− 0.259	0.027	− 0.541	0.595
Drink 2/3 days per month & 5 and more standard drinks	−1.912***	−2.526	− 1.299	− 0.501	− 1.112	0.110
Drink 1/2 days per week & 1 to 2 standard drinks	− 0.726***	− 1.212	− 0.240	0.479*	−0.011	0.969
Drink 1/2 days per week & 3 to 4 standard drinks	−1.308***	− 1.847	− 0.768	0.048	− 0.492	0.589
Drink 1/2 days per week & 5 and more standard drinks	−1.735***	−2.313	− 1.157	− 0.806***	− 1.381	− 0.231
Drink 3 or more days per week & 1 to 2 standard drinks	− 0.962***	− 1.489	− 0.435	0.301	− 0.236	0.839
Drink 3 or more days per week & 3 to 4 standard drinks	−1.896***	− 2.460	− 1.332	− 0.292	− 0.863	0.279
Drink 3 or more days per week & 5 and more standard drinks	− 2.998***	−3.664	− 2.332	−1.342***	− 1.995	− 0.689
Non-Carer: How often participate in Physical Activities?						
Not at all (Reference group)						
Less than once a week	1.304***	0.942	1.665	2.219***	1.847	2.591
1 to 2 times a week	2.412***	2.042	2.781	4.118***	3.733	4.502
3 times a week	3.394***	2.996	3.791	5.679***	5.271	6.087
More than 3 times a week	4.528***	4.131	4.926	7.628***	7.210	8.045
Non-Carer: How often get together socially with friends/relatives?						
Every Day	4.984***	4.297	5.670	3.008***	2.361	3.656
Several times a week	4.483***	3.947	5.018	2.695***	2.203	3.186
About once a week	3.434***	2.920	3.947	2.115***	1.648	2.582
1 to 3 times a month	2.536***	2.041	3.032	1.588***	1.136	2.041
1 or 2 times every 3 months	1.155***	0.607	1.703	0.501**	0.000	1.002
Less often than once every 3 months (Reference group)						
Non-Carer: Smoking Status						
[Never smoked/No longer smoke (Reference group)						
Smoke less often than weekly	0.053	−0.710	0.816	−0.836**	−1.597	− 0.076
Smoke at least weekly (but not daily)	−0.433	− 1.175	0.309	−1.175***	− 1.891	− 0.460
Smoke daily	− 0.746**	− 1.360	− 0.133	− 1.231***	− 1.838	− 0.625
Differences in Carer versus Non-carer Status: interaction terms						
Carer & Age	0.024*	− 0.004	0.051	0.034**	0.006	0.062
Carer & Time spent in (hrs/mins) per week Caring for disabled/elderly relative	0.021	−0.008	0.050	0.012	−0.013	0.036
Carer & Employment Status						
Employed - works 35h hours a week (Reference group)						
Employed - works less than 35 h a week	0.100	−0.835	1.036	−0.312	−1.195	0.571
Unemployed, retired, home duties, students & others	0.057	−0.846	0.961	−0.291	−1.184	0.602
Carer & Alcohol drinking status						
Never drink/No longer drink (Reference group)						
Drink only rarely & 1 to 2 standard drinks	0.279	−0.791	1.349	0.717	−0.331	1.766
Drink only rarely & 3 to 4 standard drinks	2.165**	0.234	4.096	1.922*	−0.132	3.977
Drink only rarely & 5 and more standard drinks	−0.577	−3.043	1.890	−0.338	−2.866	2.190
Drink 2/3 days per month & 1 to 2 standard drinks	−0.135	−1.603	1.334	0.020	−1.461	1.500
Drink 2/3 days per month & 3 to 4 standard drinks	2.389**	0.435	4.342	2.073*	−0.032	4.178
Drink 2/3 days per month & 5 and more standard drinks	−0.299	−2.747	2.149	0.465	−1.699	2.630
Drink 1/2 days per week & 1 to 2 standard drinks	0.462	−0.857	1.783	−0.222	−1.545	1.101
Drink 1/2 days per week & 3 to 4 standard drinks	1.028	−0.778	2.835	−0.375	−2.101	1.350
Drink 1/2 days per week & 5 and more standard drinks	−1.144	−3.222	0.935	0.679	−1.612	2.970
Drink 3 or more days per week & 1 to 2 standard drinks	−0.237	−1.401	0.926	0.347	−0.847	1.540
Drink 3 or more days per week & 3 to 4 standard drinks	0.340	−1.065	1.746	1.091	−0.273	2.455
Drink 3 or more days per week & 5 and more standard drinks	0.653	−1.168	2.475	0.958	−0.716	2.632
Carer & How often participate in Physical Activities?						
Not at all (Reference group)						
Less than once a week	0.419	−0.766	1.605	−0.085	−1.182	1.012
1 to 2 times a week	1.050*	−0.092	2.191	0.502	−0.584	1.589
3 times a week	1.273**	0.069	2.477	0.346	−0.817	1.509
More than 3 times a week	1.005*	−0.137	2.147	0.073	−1.037	1.182
Carer & How often get together socially with friends/relatives?						
Every Day	−1.529	−3.812	0.753	−1.016	−3.363	1.332
Several times a week	0.498	−1.032	2.027	0.633	−0.774	2.041
About once a week	−0.009	−1.480	1.462	0.289	−1.066	1.644
1 to 3 times a month	0.110	−1.312	1.532	0.453	−0.877	1.782
1 or 2 times every 3 months	1.176	−0.500	2.853	0.633	−0.932	2.199
Less often than once every 3 months (Reference group)						
Carer & Smoking Status						
Never smoked/No longer smoke (Reference group)						
Smoke less often than weekly	−1.820	−4.439	0.799	0.525	−2.155	3.204
Smoke at least weekly (but not daily)	−1.348	−4.176	1.480	0.056	−2.913	3.025
Smoke daily	0.660	−0.522	1.843	−0.382	−1.466	0.702
Other Overall Control Variables: effect for the whole sample						
Standardized Household financial year gross total income ($)	0.195***	0.091	0.298	0.129***	0.031	0.227
Life events in past year: Serious personal injury/illness	−3.713***	−4.025	−3.401	−6.182***	−6.518	−5.846
Time spent (hrs/mins) per week Volunteer/Charity work	0.026*	−0.002	0.054	0.017	−0.015	0.049
Number of cigarettes usually smoked each week/10	−0.037	−0.085	0.011	−0.050**	− 0.096	−0.005

***Notes.*** All the *p*-values have been replaced by stars and categorised as follows. ***: *p* < 0.01; **: *p* < 0.05; *: *p* < 0.1; However, confidence intervals use the usual 95% confidence level. The first row presents the impact of *Carer Status* on SF36 mental health and general health outcomes of individuals over time. The following section titled *Effects on Non-Carer Status: main terms*, presents the estimates of individual characteristics such as their age and caregiving time and the impact of their health behaviours (physical activity level, smoking and drinking level), employment status and level of social engagement on their self-assessed health status when they were *non-carers*. The next section titled *Differences in Carer* versus *Non-carer Status: interaction terms*, presents the non-carer and carer status differential estimates (the carer status has been interacted with other characteristics to estimate the magnitude of these differentials). Therefore, for each variable with both main and interaction terms in the model, the effect associated with carer status is given by the sum of both the non-carer status effect and the differential effects. The last section titled *Other Overall Control Variables: effect for the whole sample* presents the effects of characteristics associated to the whole sample (effects assumed to be the same in both non-care and carer groups)

**Table 4:** Estimates of the Fixed Effects Base Model for SF36 Physical Functioning Component

Variables	SF36 Physical Functioning Component		
	Coef. (Standard Error)	[95% Conf. Coef.	
Carer Status (Yes/No): Actively cares for a household member/non-resident due to long-term health condition, elderly	− 0.199 (0.240)	−0.669	0.271
Constant	83.913*** (.017)	83.880	83.946
sigma_u	21.537		
sigma_e	13.109		
rho	0.730 (fraction of variance due to u_i)		

Notes. All the p-values have been replaced by stars and categorised as follows. ***: p < 0.01; **: p < 0.05; *: p < 0.1; However, confidence intervals use the usual 95% confidence level; sigma_u: Within-individuals variance; sigma_e: Residual variance; rho: Percentage of total variance due to within-individuals variation (or ICC, the intra-class correlation, measure of intra-individual correlation)

### Aging and Carer health

**Fig. 3:**
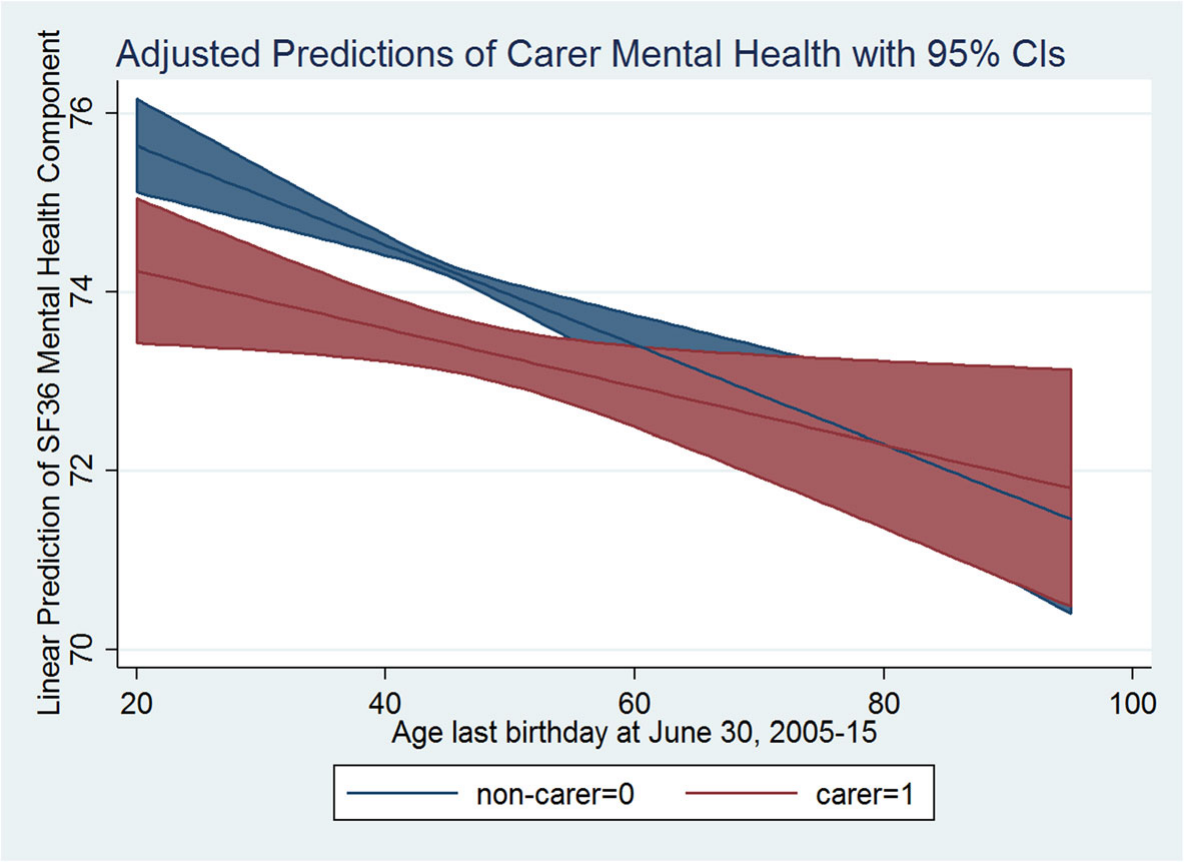
Adjusted Predictions of Carer Mental Health with 95% CIs

**Fig. 4:**
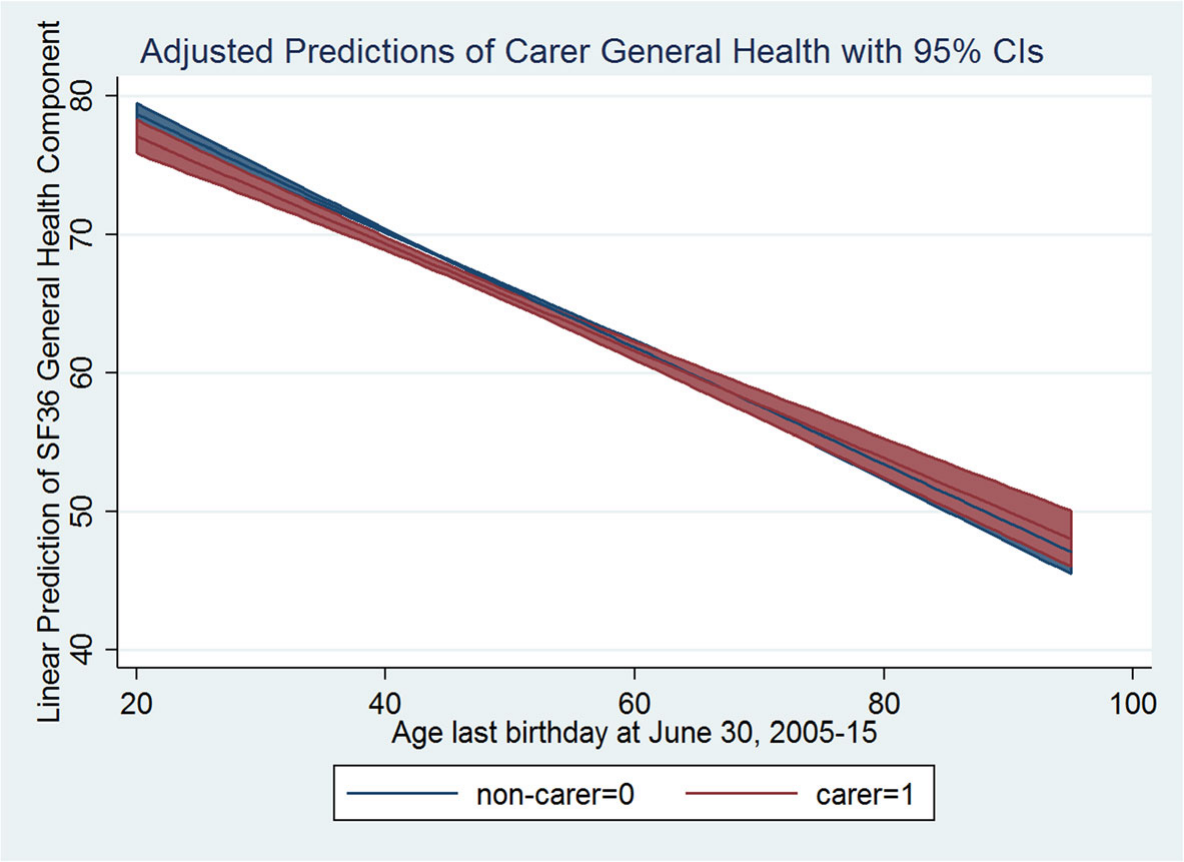
Adjusted Predictions of Carer General Health with 95% CIs

The carer health trends over time revealed interesting patterns in this study. There was a clear pattern of decreasing mental and general health status for both carers and non-carers with their age. Figures 3 and 4 present mental and general health marginal mean trends respectively (adjusted predictions) over time (with age as time) for carer and non-carer groups. Both mental and general health scores decreased significantly with age, but at different rates for carers and non-carers. In unadjusted models for mental health, estimates of rates of change were: Beta = − 0.054 (95% CI: (− 0.084, − 0.024), *p* < 0.001) for non-carers; Beta = − 0.034 (95% CI: (− 0.071, 0.004), *p* = 0.077) for carers, and the difference in rates of change was not statistically significant (*p* = 0.088). For general health, estimates were: Beta = − 0.477 (95% CI: (− 0.509, − 0.443), p < 0.001); Beta = − 0.437 (95% CI: (− 0.477, − 0.397), p < 0.001), for non-carers and carers respectively, and the difference in slopes was significant (*p* = 0.002). As shown in Figs. 3 and 4, carer status modified the effect of aging on mental and general health with a slower rate of decline for carers compared to non-carers (moderation effect). The decreasing pattern in mental and general health with aging remained for non-carer status, in fully adjusted models as shown in Table 3. However, the adjusted decrease was non-significant for mental health, but significant for general health. The difference in rates of change due to carer status was on the margin of statistical significance for mental health at the 10% level, but remained significant for general health at 5% level.

### Health behaviour and Carer health

Interactions of individuals’ health behaviours, such as their drinking and smoking status and physical activity level, with their carer/non-carer status, revealed quite intriguing relations with both mental and general health scores.

Results for alcohol drinking status when interacted with the number of standard drinks consumed per day revealed that in non-carers, any level of drinking, compared to those who did not drink, had a negative effect on mental health score with increasing patterns. However, the drinking status exhibited quite an intriguing pattern in terms of its effect on non-carers’ general health score (Table 3, 4^th^ column). For example, if a person was a social drinker and drank only 2–3 days per month with only 1 to 2 standard drinks or even 1–2 days per week with 1 to 2 standard drinks then that had a positive effect on general health score compared to those who did not drink. On the other hand, if a person drank 5 or more standard drinks for only 2 to 3 days per month or drank on a weekly basis then that had a negative effect on general health score. In the case of carer status, the effect modification was significant for mental health only in light drinkers (“drink only rarely”, or “drink 2-3 days per month”) who consumed 3 to 4 standard drinks with on average a 2-points higher score. The same effect modification was apparent for general health in the same groups as for mental health. However, the 2-points difference on average (in favour of carers) was only marginally significant at the 10% level.

As expected for smoking status, results revealed that, in non-carers, smoking daily had a significant negative effect on mental health score, whereas any level of smoking had a negative effect on general health score, increasing with the level of smoking status. For those in carer status, light (smoke less often than weekly) and medium (smoke at least weekly, but not daily) smoking was more detrimental to mental health than was smoking daily. This was not the case for general health, where the negative effect of smoking got stronger as the levels of smoking increased. Overall, we have also controlled for the number of cigarettes a person usually smokes per week and found a statistically significant negative effect on the persons’ general health (irrespective of carer/non-carer status).

As for physical activity status, when someone was a non-carer, any level of exercising, compared to those who did not undertake any activity, had a positive and increasing influence on both mental and general health scores, with more activities generating better health outcomes. Carer/non-carer status differences in effects appeared to be all positive for mental health, meaning that physical activities were even more beneficial when someone served as a carer, though the benefit was significant only at the physical activity level of 3 times per week.

### Social engagement, employment and Carer health

Overall, the level of social engagement and employment status when serving as a carer did not show any significant carer and non-carer status differential effect on health. However, results on employment status revealed that a non-carer and full-time employed person reported both better mental and general health scores compared to part-time employees, pensioners, students and housewives. But, employment status had no significant modifying effect on mental and general health scores when someone served as a carer.

Similarly, for social interactions and communication with friends and relatives, when non-carer, the more often a person got together with friends/relatives, compared to infrequent interactions (less often than once in 3 months), the better mental and general health status they enjoyed. However, results showed no significant carer/non-carer status differences.

## Discussion

This paper presents a large population-based retrospective study of individual level mental and general health of Australians in relation to their informal caregiving status using a longitudinal analysis. The study added substantial value to the literature on carer’s health by conducting a general population-based analysis that follows the individual person over their pre-to-post caregiving years. The study supported the evidence that being an active carer for a household member/non-resident due to long-term health condition and/or elderly status had a negative impact on both mental and general health of the carer. The time spent in care giving had a negative effect on mental health even for non-active carers when they only spent a few hours on caregiving tasks per week. Further, the study identified modifying effects of some of the risk and protective factors for carer health in Australia.

### Aging and Carer health

The aging of an individual is associated with worsening physical and/or mental health status in the literature [4, 48]. Mental health may in turn be influenced by the reduction in physical health (or vice versa) and loss of physical functioning due to aging and associated disability that renders a person less mobile. This effect was evidenced in this study with a significant negative effect of age on general health score when someone was a non-carer and a negative (but non-significant) effect on mental health. On the contrary, general population level epidemiological research suggests that the prevalence of mental illness decreases considerably with increasing age. There is an understanding that the prevalence of mental distress is highest in the 25–34 age group and decreases with increasing age [49]. Nevertheless, this study investigated the influence of caregiving status on an individual’s health over their life (following the individual over time) and led to the observation of a protective effect of age on carer’s health. In doing so, as documented earlier in this paper, the individual person’s initial health status was an important confounder in the analysis. Consequently, adjusted predictions with age in Figs. 3 and 4 supported the evidence that, at the individual level for non-carers, irrespective of their initial (general/mental) health status, both general and mental health deteriorated with age. More importantly, the study found a positive impact of caregiving status through the attenuation of the carer’s age effect on both mental and general health. This is an important finding that has significant policy implication. In case of carers, increasing age is associated with less decline in health. With age, carers report better mental and general health than non-carers. In other words, carer and non-carer differences in health may be less pronounced in older age than in younger age. It may also be the case that after certain age, older carers exhibit even better health outcomes than non-carers of the same age. This finding may help policy makers to design interventions to support young carers. Young informal carers may find themselves prematurely burdened with the unprecedented caregiving needs of their loved ones. In substantiating our findings, some recent Australian studies have recognised that the young informal carers are indeed at disadvantage compared to carers in older age groups. They face reduced opportunities to access education and employment, or to participate in social and community activities and consequently may also be experiencing financial hardship [50–53]. An important policy initiative to improve their circumstances, may be to provide better income support that could allow them additional time and resources needed. Community programs involving interactive social and physical activities could help young carers to cope better with their caregiving burden. Additionally, respite care programs may be more productive if focused on young carers [49]. Most informal carers in Australia, however, are in the working age group (25–64 years) and the average carers’ age across waves in our sample is 52 years. Carers in this age group, while facing similar difficulties and benefits as carers in other age groups in terms of reduced participation in the labour force, increased unemployment and reduced earnings, they also face the extra burden of needing and/or wanting to work, or to remain in education or training, while providing care to others [50, 51]. On the other hand, in Australia evidence supports that older carers’ circumstances are somehow better than others. They are likely to be more experienced as carers, their income and social support network are less likely to be affected by their caregiving responsibilities, while they also tend to rather enjoy their caregiving role [50, 51]. Therefore, the positive attenuation effect of age on carer general and mental health in this paper is rather a robust finding that should be supported with appropriate policy and used for designing age-specific interventions.

### Health behaviour and Carer health

Consistent with existing literature on the positive relationship between physical activity and cardio-metabolic health [54–57], this study found that physical activity had a positive and significant influence on mental and general health when someone was a non-carer. More importantly, physical activities also had an additional modifying effect on mental health when someone was a carer. Based on previous research, while carers may predispose themselves into sedentary behaviour [58], this study found that, while serving as a carer, pursuing physical activities had a protective beneficial impact on their mental health over and above that observed for non-carers. This finding suggests that designing tailored therapeutic physical activity interventions should be considered to improve carers’ mental health, and may also support their overall general health [58].

As expected, the results revealed that when people were non-carers, any level of alcohol consumption was harmful for their mental health, while a high level of alcohol consumption was harmful for their general health. However, one intriguing finding of this research was that some level of social drinking had a beneficial modifying impact on both mental and general health when people served as carers. This finding runs counter to non-carer drinking behaviours’ effects on health. Previous research supports that caregivers who experience social and emotional burden related to caregiving are at risk for problematic alcohol use that may need mental health and public health support [59, 60]. However, there is also evidence that carers, sometimes, take alcohol in order to forget their burden of caregiving and get some relief as a coping strategy. For that reason, some level of social drinking may have beneficial health effects on them [59, 60]. While our research suggests, with caution, that social drinking may be promoted as a therapeutic intervention to support carer health, we also identify the need for further research to better understand the link between carer social drinking habits and their health.

Further, this research supported that smoking had a negative impact on non-carers’ mental and general health while the effect was even worse when people served as carers. For carers, the results suggested that light (smoke less often than weekly) and medium (smoke at least weekly (but not daily)) smoking were more detrimental to mental health than daily smoking. This led to our assertion that smoking here may have been used as a coping mechanism as there is previous evidence that smoking is used as a coping mechanism for psychological stress [61].

### Social engagement, employment and Carer health

Literature on carer’s employment status and health have identified that high intensity caregiving does not go well with full-time employment in Australia or even allows no-workforce engagement such as in Japan [22, 25]. However, this study found no significant modifying impact of employment on carers’ health. Furthermore, in a separate model, when we included high-intensity and low-intensity caregiving categories based on their caregiving hours and interacted that with carer employment participation, we found no modifying impact either on their mental or general health.

In line with previous literature, this research found that social interaction and community participation had significant positive impacts on non-carers mental and general health [27–30, 62]. However, we did not find any significant attenuation effect of these activities on health when people served as carers. Whilst we found that social interactions (not significant) might not be beneficial for carers’ mental health on a daily/weekly basis, they might prove beneficial when considered on a monthly/quarterly basis.

### Summarizing the discussion

To sum up, this study added value to the literature on informal carers’ mental and general health in Australia by identifying potential protective and risk factors. Using 11 waves of HILDA data and FE regression approach with panel-robust standard errors, the study found modifying effects of carers’ health behaviours such as physical activity, smoking and drinking status while handling the issue of individuals self-selecting themselves as carers due to some predisposing factors such as socioeconomic status and sedentary behaviours. More importantly, the protective effects of physical activity and the possible benefits of socialising with alcohol (although this is not supported by the findings around social contacts with family and friends) need to be highlighted with caution for designing potential interventions.

Previous research found carers showing better fitness outcomes on specific components of their physical functioning than others [11]. We conducted separate analysis of carer physical functioning but found no evidence of a relation between caregiving status and physical functioning level.

The strength of this study lies in applying advanced econometric/statistical techniques to the already existing longitudinal, data set (HILDA). The study used a retrospective longitudinal design and the “fixed-effects” modelling approach that allowed to net out the effects of caregiving outcomes over the lifetime of an individual while adjusting for other time-variant potential confounders. The weaknesses include the observational nature of the survey data as opposed to controlled randomized design and the use of individual level characteristics only in the FE regression while there may be contextual variables shaping individual trajectories of mental and general health outcomes. Also, there may be issues with potentially low representativeness of the study sample to Australian population. Being a longitudinal study design, the response rates in the study would be low compared to cross-sectional surveys and decline with time due to attrition. It is highly likely that certain groups of the Australian population such as immigrants, Culturally and Linguistically Diverse (CALD) communities and low socio-economic status families may have low representation in the survey. Another limitation is the use of self-reported information such as caregiving status and outcome variables such as SF-36 mental and general health variables. The SF-36 mental health (SF36MH) and general health (SF36GH) have been widely used and extensively validated with reported excellent psychometric properties, although they may not necessarily be validated for Australian migrant or CALD subpopulations.

## Conclusion

This study has made some notable contributions in understanding the health of Australian informal carers. The research indicates that poorer mental and general health profile of carers compared to non-carers in Australia can potentially be improved through targeted support programs for young carers. Additionally, targeted physical activity intervention programs may have beneficial mental health effects on carers. The potential link between moderate level of social drinking and carer health identified needs to be further explored with more targeted future research. Studies like this and suggested interventions would help care recipients and the greater society. Indeed, improved carer health may help to reduce the formal/institutional demand for carers and could bridge the gap in carers demand and supply.

## Data Availability

This paper uses unit record data from the Household, Income and Labour Dynamics in Australia (HILDA) Survey. The HILDA survey data is one of the Australian Government Department of Social Services (DSS) longitudinal datasets housed by the National Centre for Longitudinal Data (NCLD) and managed by the Australian Data Archive (ADA). The datasets analysed and/or generated during the current study are subject to the Confidentiality Deed signed with the Commonwealth of Australia (as represented by the Department of Social Services) and to the Commonwealth privacy laws. Data are accessible from the NCLD by application (https://www.dss.gov.au/national-centre-for-longitudinal-data-ncld/access-to-dss-longitudinal-datasets), and any questions about applying for the DSS longitudinal datasets should be addressed to NCLD (ncld@dss.gov.au).

## Abbreviations

ADL: Activities of Daily Living
CALD: Culturally and Linguistically Diverse
FE: Fixed Effects
HILDA: Household Income and Labour Dynamics of Australia
IQOLA: International Quality of Life Assessment
RE: Random Effects
SCQ: Self-completion Questionnaire
SD: Standard Deviation
SF-36: Short Form 36 Questionnaire
SF36GH: SF-36 General Health
SF36MH: SF-36 Mental Health
